# A guide to selecting high-performing antibodies for amyloid-beta precursor protein for use in Western Blot, immunoprecipitation and immunofluorescence

**DOI:** 10.12688/f1000research.139867.2

**Published:** 2024-04-10

**Authors:** Riham Ayoubi, Maryam Fotouhi, Donovan Worrall, Kathleen Southern, Carl Laflamme, NeuroSGC/YCharOS/EDDU collaborative group, ABIF consortium

**Affiliations:** 1 Department of Neurology and Neurosurgery, Structural Genomics Consortium, The Montreal Neurological Institute, McGill University, Montreal, Québec, H3A 2B4, Canada

**Keywords:** Uniprot ID P05067, APP, Amyloid-beta precursor protein, antibody characterization, antibody validation, Western Blot, immunoprecipitation, immunofluorescence

## Abstract

The amyloid-beta precursor protein is a transmembrane protein expressed in many tissues and highly concentrated in the brain. The protein is of significant interest due to its involvement in the generation of amyloidogenic β-amyloid peptides, prone to plaque formation that is characteristic of Alzheimer’s Disease. The scientific community would benefit from the availability of high-quality anti-amyloid-beta precursor protein antibodies to enhance reproducible research on this target. In this study, we characterized eleven amyloid-beta precursor protein commercial antibodies for Western blot, immunoprecipitation, and immunofluorescence using a standardized experimental protocol based on comparing read-outs in knockout cell lines and isogenic parental controls. These studies are part of a larger, collaborative initiative seeking to address antibody reproducibility issues by characterizing commercially available antibodies for human proteins and publishing the results openly as a resource for the scientific community. While use of antibodies and protocols vary between laboratories, we encourage readers to use this report as a guide to select the most appropriate antibodies for their specific needs.

## Introduction

The amyloid-beta precursor protein, encoded by the *APP* gene, is a transmembrane protein with a single transmembrane domain, a large extracellular ectodomain and a short cytoplasmic tail. ^1^ Although ubiquitously expressed, amyloid-beta precursor protein is predominantly found on the surface of neurons, where it promotes neurite growth, neuronal adhesion and axonogenesis. ^2^

Discovered and isolated in the 1980’s, amyloid-beta precursor protein has become a significant area of interest as its proteolytic cleavage can give rise to amyloid β-proteins (Aβ). ^1^^,^^3^^,^^4^ Aβ are small 40-42 amino acid-long peptides related to the pathogenesis of Alzheimer’s Disease as their accumulation can result in the formation of senile amyloid plaques. ^1^^,^^5^ Further research is required to elucidate the pathway underlying the proteolytic processing of amyloid-beta precursor protein and in turn determine its potential as a diagnostic marker or therapeutic target to prevent degenerative brain progression. Mechanistic studies would be greatly facilitated with the availability of high-quality antibodies. This research is part of a broader collaborative initiative in which academics, funders and commercial antibody manufacturers are working together to address antibody reproducibility issues by characterizing commercial antibodies for human proteins using standardized protocols, and openly sharing the data. ^6^^–^^8^

In this study, we compared the performance of eleven commercially-available antibodies for amyloid-beta precursor protein for use in Western blot, immunoprecipitation and immunofluorescence, enabling biochemical and cellular assessment of amyloid-beta precursor protein’s properties and function.

## Results and discussion

Our standard protocol involves comparing readouts from wild-type (WT) and knockout (KO) cells. ^9^^–^^19^ The first step was to identify a cell line(s) that expresses sufficient levels of a given protein to generate a measurable signal. To this end, we examined the DepMap transcriptomics database to identify all cell lines that express the target at levels greater than 2.5 log _2_ (transcripts per million “TPM” + 1), which we have found to be a suitable cut-off (Cancer Dependency Map Portal, RRID:SCR_017655). Commercially available HAP1 cells expressed the amyloid-beta precursor protein transcript at RNA levels above the average range of cancer cells analyzed. Parental and *APP* KO HAP1 cells were obtained from Horizon Discovery ( Table 1).

**Table 1.** Summary of the cell lines used.

Institution	Catalog number	RRID (Cellosaurus)	Cell line	Genotype
Horizon Discovery	C631	CVCL_Y019	HAP1	WT
Horizon Discovery	HZGHC005368c010	CVCL_SD04	HAP1	APP KO

**Figure 1.**
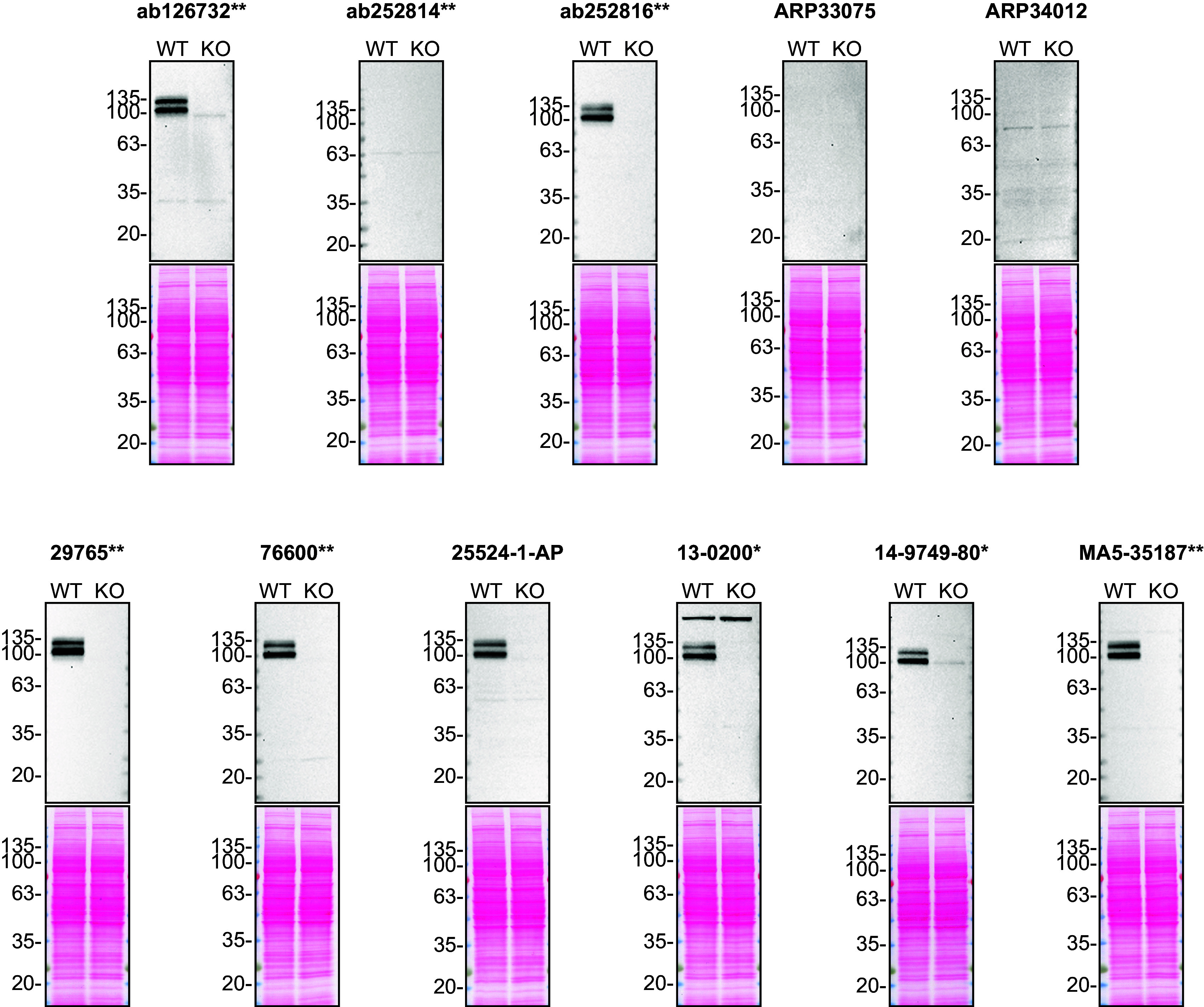
Amyloid-beta precursor protein antibody screening by Western Blot. Lysates of HAP1 (WT and *APP* KO) were prepared and 50 μg of protein were processed for Western Blot with the indicated Amyloid-beta precursor protein antibodies. The Ponceau stained transfers of each blot are presented to show equal loading of WT and KO lysates and protein transfer efficiency from the acrylamide gels to the nitrocellulose membrane. Antibody dilutions were chosen according to the recommendations of the antibody supplier. Exceptions were given for antibodies ab126732**, 29765** and 76600** which were all titrated to 1/500, as the signals were too weak when following the supplier’s recommendations. Antibody dilution used: ab126732** at 1/500, ab252814** at 1/500, ab252816** at 1/500, ARP33075 at 1/500, ARP34012 at 1/500, 29765** at 1/500, 76600** at 1/500, 25524-1-AP at 1/500, 13-0200* at 1/200, 14-9749-80* at 1/500, MA5-35187** at 1/500. Predicted band size: 87 kDa. *Monoclonal antibody, **Recombinant antibody.

For Western Blot experiments, we resolved proteins from WT and *APP* KO cell extracts and probed them side-by-side with all antibodies in parallel ( Figure 1). ^10^^–^^19^

**Figure 2.**
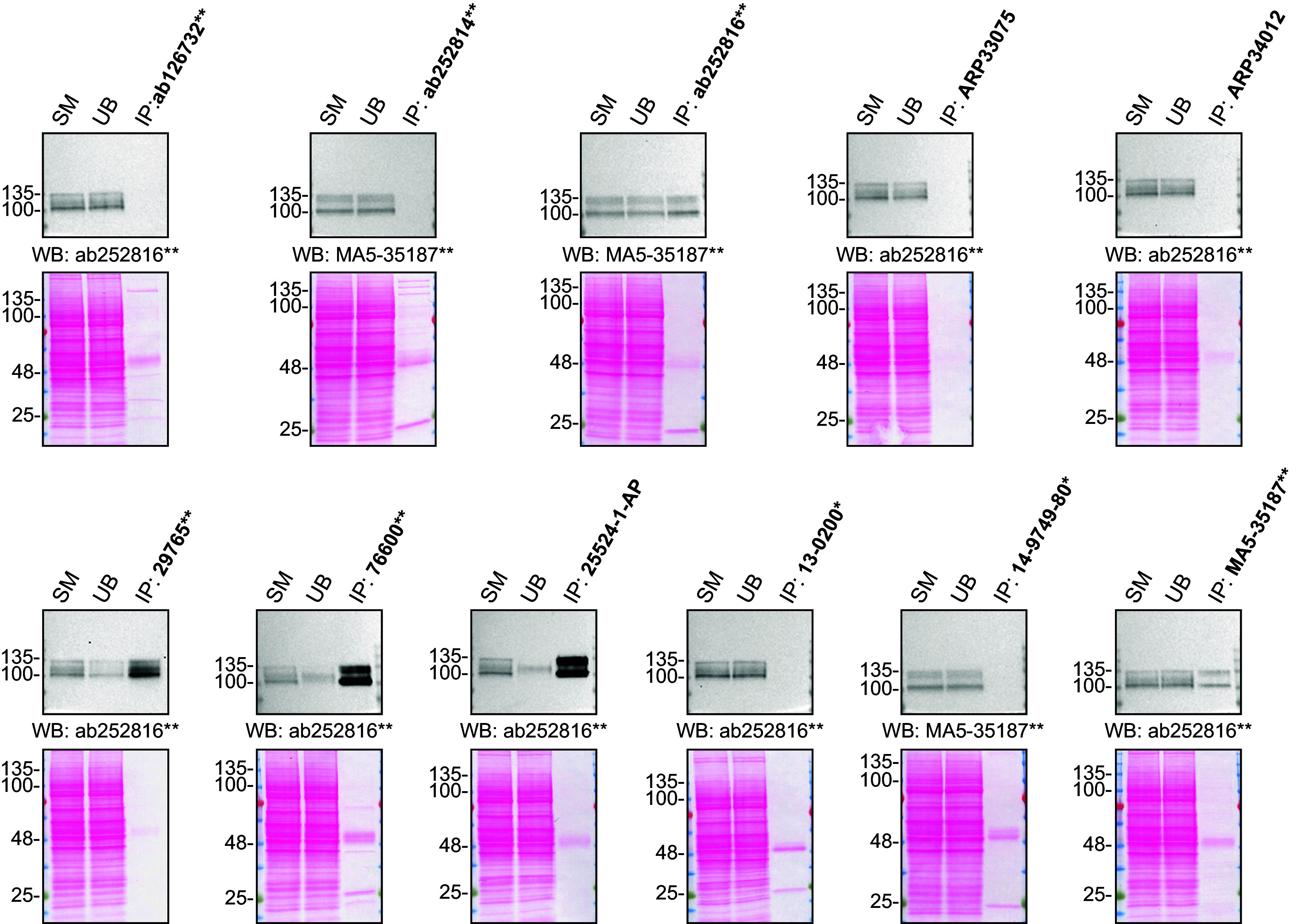
Amyloid-beta precursor protein antibody screening by immunoprecipitation. HAP1 lysates were prepared, and IP was performed using 2.0 μg of the indicated Amyloid-beta precursor protein antibodies pre-coupled to Dynabeads protein G or protein A. Samples were washed and processed for Western Blot with the indicated Amyloid-beta precursor protein antibodies. For Western Blot, MA5-35187** and ab252816** were used at 1/500. The Ponceau stained transfers of each blot are shown for similar reasons as in Figure 1. SM=4% starting material; UB=4% unbound fraction; IP=immunoprecipitate, *Monoclonal antibody, **Recombinant antibody.

For immunoprecipitation experiments, we used the antibodies to immunopurify amyloid-beta precursor protein from HAP1 cell extracts. The performance of each antibody was evaluated by detecting amyloid-beta precursor protein in extracts, in the immunodepleted extracts and in the immunoprecipitates ( Figure 2). ^10^^–^^19^

**Figure 3.**
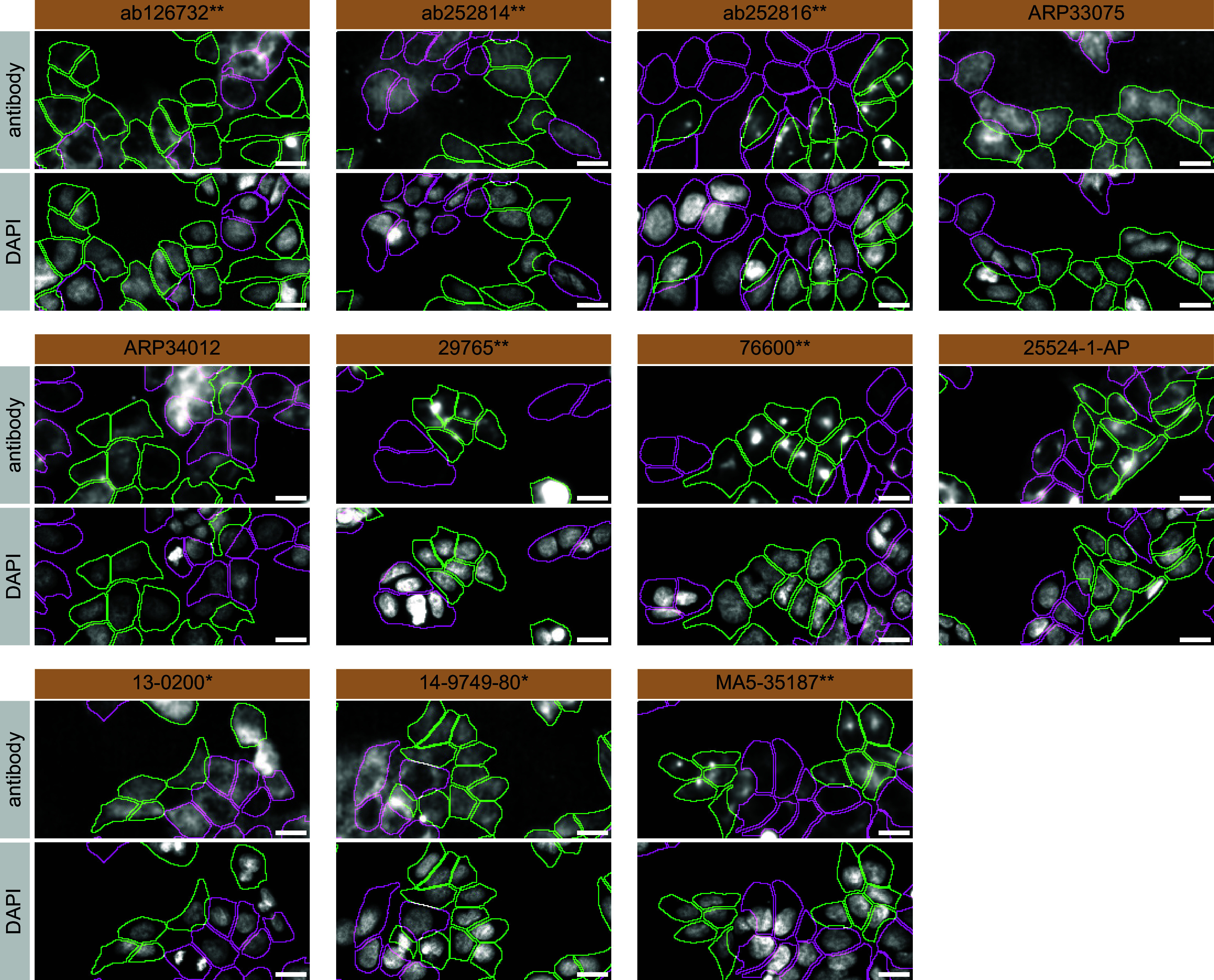
Amyloid-beta precursor protein antibody screening by immunofluorescence. HAP1 WT and *APP* KO cells were labelled with a green or a far-red fluorescent dye, respectively. WT and KO cells were mixed and plated to a 1:1 ratio in a 96-well plate with an optically clear flat-bottom. Cells were stained with the indicated Amyloid-beta precursor protein antibodies and with the corresponding Alexa-fluor 555 coupled secondary antibody including DAPI. Acquisition of the blue (nucleus-DAPI), green (WT), red (antibody staining) and far-red (KO) channels was performed. Representative images of the merged blue and red (grayscale) channels are shown. WT and KO cells are outlined with green and magenta dashed line, respectively. When the concentration was not indicated by the supplier, we tested antibodies at 1/100 and 1/500. At these concentrations, the signal from each antibody was in the range of detection of the microscope used. Antibody dilution used: ab126732** at 1/500, ab252814** at 1/500, ab252816** at 1/500, ARP33075 at 1/500, ARP34012 at 1/500, 29765** at 1/100, 76600** at 1/50, 25524-1-AP at 1/500, 13-0200* at 1/500, 14-9749-80* at 1/500, MA5-35187** at 1/1000. *Monoclonal antibody, **Recombinant antibody, Bars=10 μm.

For immunofluorescence, antibodies were screened using a mosaic strategy. ^20^ First, HAP1 WT and *APP* KO cells were labelled with different fluorescent dyes in order to distinguish the two cell lines, and the eleven amyloid-beta precursor protein antibodies were evaluated by immunofluorescence. Cells were imaged in the same field of view to reduce staining, imaging and image analysis bias ( Figure 3). Quantification of immunofluorescence intensity in hundreds of WT and KO cells was performed for each antibody tested. The images presented in Figure 3 are representative of the results of this analysis.

In conclusion, we have screened eleven amyloid-beta precursor protein commercial antibodies by Western blot, immunoprecipitation and immunofluorescence. Several high-quality antibodies that successfully detect amyloid-beta precursor under our standardized experimental conditions can be identified. In our effort to address the antibody reliability and reproducibility challenges in scientific research, the authors recommend the antibodies that demonstrated to be underperforming under our standard procedure be removed from the commercial antibody market. However, the authors do not engage in result analysis or offer explicit antibody recommendations. A limitation of this study is the use of universal protocols – any conclusions remain relevant within the confines of the experimental setup and cell line used in this study. Our primary aim is to deliver top-tier data to the scientific community, grounded in Open Science principles. This empowers experts to interpret the characterization data independently, enabling them to make informed choices regarding the most suitable antibodies for their specific experimental needs. An editorial by Biddle et al. provides valuable insights on how to interpret the antibody characterization data found in this article. ^21^ The underlying data can be found on the Zenodo open access repository. ^22^^,^^23^

## Methods

### Antibodies

All amyloid-beta precursor protein antibodies are listed in Table 2, together with their corresponding Research Resource Identifiers, or RRID, to ensure the antibodies are cited properly. ^24^ Peroxidase-conjugated goat anti-mouse, anti-mouse and anti-rat antibodies are from Thermo Fisher Scientific (cat. number 62-6520, 65-6120 and 31470, respectively). Alexa-555-conjugated goat anti-mouse and anti-rabbit secondary antibodies are from Thermo Fisher Scientific (cat. number A21424 and A21429).

**Table 2.** Summary of the amyloid-beta precursor protein antibodies tested.

Company	Catalog number	Lot number	RRID (Antibody Registry)	Clonality	Clone ID	Host	Concentration (μg/μL)	Vendors recommended applications
Abcam	ab126732 **	GR34478901	AB_11131727	recombinant-mono	EPR5118-34	rabbit	0.114	Wb
Abcam	ab252814 **	GR34013051	AB_2925224 ^1^	recombinant-mono	14D6	rat	1.154	Wb
Abcam	ab252816 **	GR33342983	AB_2925223 ^1^	recombinant-mono	2E9	rat	0.583	Wb, IP
Aviva Systems Biology	ARP33075	QC3561-90401	AB_2044934	polyclonal	-	rabbit	0.5	Wb
Aviva Systems Biology	ARP34012	QC47046-42571	AB_2044933	polyclonal	-	rabbit	0.5	Wb
Cell Signaling Technology	29765 **	1	AB_2925221 ^1^	recombinant-mono	E8B3O	rabbit	n/a	Wb, IP
Cell Signaling Technology	76600 **	1	AB_2925222 ^1^	recombinant-mono	E4H1U	rabbit	n/a	Wb, IP
Proteintech	25524-1-AP	51555	AB_2880118	polyclonal	-	rabbit	0.55	Wb, IF
Thermo Fisher Scientific	13-0200 *	XC339604	AB_2532993	monoclonal	LN27	mouse	0.5	Wb, IP, IF
Thermo Fisher Scientific	14-9749-80 *	2484403	AB_2572977	monoclonal	22C11	mouse	0.5	Wb, IF
Thermo Fisher Scientific	MA5-35187 **	XH3670292	AB_2849091	recombinant-mono	ARC0465	rabbit	0.93	Wb, IF

Wb=Western blot; IF=Immunofluorescence; IP=Immunoprecipitation.

* Monoclonal antibody.

** Recombinant antibody.

^1^ Refers to RRID recently added to the Antibody Registry (in May 2023), they will be available on the Registry website in coming weeks.

### Cell culture

Both HAP1 WT and *APP* KO cell lines used are listed in Table 1, together with their corresponding RRID, to ensure the cell lines are cited properly. ^25^ Cells were cultured in DMEM high glucose (GE Healthcare cat. number SH30081.01) containing 10% fetal bovine serum (Wisent, cat. number 080450), 2 mM L-glutamate (Wisent cat. number 609065, 100 IU penicillin) and 100 μg/mL streptomycin (Wisent cat. number 450201).

### Antibody screening by Western Blot

Western Blots were performed as described in our standard operating procedure. ^26^ HAP1 WT and *APP* KO were collected in RIPA buffer (25 mM Tris-HCl pH 7.6, 150 mM NaCl, 1% NP-40, 1% sodium deoxycholate, 0.1% SDS) supplemented with 1× protease inhibitor cocktail mix (MilliporeSigma, cat. number 78429). Lysates were sonicated briefly and incubated for 30 min on ice. Lysates were spun at ~110,000 × g for 15 min at 4°C and equal protein aliquots of the supernatants were analyzed by SDS-PAGE and Western Blot. BLUelf prestained protein ladder (GeneDireX, cat. number PM008-0500) was used.

Western Blots were performed with precast midi 4-20% Tris-Glycine polyacrylamide gels (Thermo Fisher Scientific, cat. number WXP42012BOX) ran with Tris/Glycine/SDS buffer (bio-Rad, cat. number 1610772), loaded in Laemmli loading sample buffer (Thermo Fisher Scientific, cat. number AAJ61337AD) and transferred on nitrocellulose membranes. Proteins on the blots were visualized with Ponceau S staining (Thermo Fisher Scientific, cat. number BP103-10) which was scanned to show together with individual Western blot. Blots were blocked with 5% milk for 1 h, and antibodies were incubated overnight at 4°C with 5% milk in TBS with 0.1% Tween 20 (TBST) (Cell Signalling Technology, cat. number 9997). Following three washes with TBST, the peroxidase conjugated secondary antibody was incubated at a dilution of ~0.2 μg/mL in TBST with 5% milk for 1 h at room temperature followed by three washes with TBST. Membranes were incubated with Pierce ECL (Thermo Fisher Scientific, cat. number 32106) prior to detection with the iBright CL1500 Imaging System (Thermo Fisher Scientific, cat. number A44240).

### Antibody screening by immunoprecipitation

Immunoprecipitation was performed as described in our standard operating procedure. ^27^ Antibody-bead conjugates were prepared by adding 2 μg or 20 μL of antibody at an unknown concentration to 500 μL of Pierce IP Lysis Buffer (Thermo Fisher Scientific, cat. number 87788) in a 1.5 mL microcentrifuge tube, together with 30 μL of Dynabeads protein A - (for rabbit antibodies) or protein G - (for mouse and rat antibodies) (Thermo Fisher Scientific, cat. number 10002D and 10004D, respectively). Tubes were rocked for ~2 h at 4°C followed by two washes to remove unbound antibodies.

HAP1 WT were collected in Pierce IP buffer (25 mM Tris-HCl pH 7.4, 150 mM NaCl, 1 mM EDTA, 1% NP-40 and 5% glycerol) supplemented with protease inhibitor. Lysates were rocked for 30 min at 4°C and spun at 110,000 × g for 15 min at 4°C. 0.5 mL aliquots at 2.0 mg/mL of lysate were incubated with an antibody-bead conjugate for ~2 h at 4°C. The unbound fractions were collected, and beads were subsequently washed three times with 1.0 mL of IP lysis buffer and processed for SDS-PAGE and Western blot on precast midi 4-20% Tris-Glycine polyacrylamide gels.

### Antibody screening by immunofluorescence

Immunofluorescence was performed as described in our standard operating procedure. ^10^^–^^20^ HAP1 WT and *APP* KO were labelled with a green and a deep red fluorescence dye, respectively (Thermo Fisher Scientific, cat. number C2925 and C34565). The nuclei were labelled with DAPI (Thermo Fisher Scientific, cat. number D3571) fluorescent stain. WT and KO cells were plated on glass coverslips as a mosaic and incubated for 24 hrs in a cell culture incubator at 37°C, 5% CO _2_. Cells were fixed in 4% paraformaldehyde (PFA) (Beantown chemical, cat. number 140770-10ml) in phosphate buffered saline (PBS) (Wisent, cat. number 311-010-CL) for 15 min at room temperature and then washed three times with PBS. Cells were permeabilized in PBS with 0,1% Triton X-100 (Thermo Fisher Scientific, cat. number BP151-500) for 10 min at room temperature and blocked with PBS with 5% bovine serum albumin (BSA) (Wisent, cat. number 800-095), 5% goat serum (Gibco, cat. number 16210-064) and 0.01% Triton X-100 for 30 min at room temperature. Cells were incubated with IF buffer (PBS, 5% BSA, 0,01% Triton X-100) containing the primary Amyloid-beta precursor protein antibodies overnight at 4°C. Cells were then washed 3 × 10 min with IF buffer and incubated with corresponding Alexa Fluor 555-conjugated secondary antibodies in IF buffer at a dilution of 1.0 μg/mL for 1 h at room temperature with DAPI. Cells were washed 3 × 10 min with IF buffer and once with PBS.

Images were acquired on an ImageXpress micro widefield high-content microscopy system (Molecular Devices), using a 20x/0.95 NA water objective lens and scientific CMOS camera (16-bit, 1.97 mm field of view), equipped with 395, 475, 555 and 635 nm solid state LED lights (Lumencor Aura III light engine) and bandpass emission filters (432/36 nm, 520/35 nm, 600/37 nm and 692/40 nm) to excite and capture fluorescence emission for DAPI, CellTrackerTM Green, Alexa fluor 555 and CellTrackerTM Red, respectively. Images had pixel sizes of 0.68 × 0.68 microns. Exposure time was set with maximal (relevant) pixel intensity ~80% of dynamic range and verified on multiple wells before acquisition. Since the IF staining varied depending on the primary antibody used, the exposure time was set using the most intensely stained well as reference. Frequently, the focal plane varied slightly within a single field of view. To remedy this issue, a stack of three images per channel was acquired at a z-interval of 4 microns per field and best focus projections were generated during the acquisition (MetaExpress v6.7.1, Molecular Devices). Segmentation was carried out on the projections of CellTrackerTM channels using CellPose v1.0 on green (WT) and far-red (KO) channels, using as parameters the ‘cyto’ model to detect whole cells, and using an estimated diameter tested for each cell type, between 15 and 20 microns. ^28^ Masks were used to generate cell outlines for intensity quantification. Figures were assembled with Adobe Photoshop (version 24.1.2) to adjust contrast then assembled with Adobe Illustrator (version 27.3.1).

## Data Availability

Zenodo: Antibody Characterization Report for Amyloid-beta precursor protein, https://doi.org/10.5281/zenodo.7971926. ^22^ This project contains the following underlying data;
- Amyloid-beta precursor protein_APP_YCharOS report.pdf Zenodo: Dataset for the Amyloid-beta precursor protein antibody screening study, https://doi.org/10.5281/zenodo.8140410. ^23^ Data are available under the terms of the Creative Commons Attribution 4.0 International license (CC-BY 4.0).
