# Supplementary material for: A guide to selecting high-performing antibodies for amyloid-beta precursor protein for use in Western Blot, immunoprecipitation and immunofluorescence
Source: F1000Res. 2024 Apr 10;12:956. Originally published 2023 Aug 9. [Version 2] doi: 10.12688/f1000research.139867.2 (PMC11445640; doi:10.12688/f1000research.139867.2)
Supplement: Peer review history

# Reviewer report: Reviewer response for version 2

**DOI:** 10.5256/f1000research.164529.r326943

**Published:** 2024-10-11

**Authors:** Karen Bowman

**Affiliations:** 1 University of Leicester, Leicester, England, UK

The study involved characterization of eleven commercial antibodies, from five different suppliers, for the amyloid-beta precursor protein (APP) for use in western blot, immunoprecipitation, and immunofluorescence. The study formed part of the wider YCharOS collaboration to characterize commercial antibodies for human proteins using standardized protocols with the intention of improving antibody reproducibility issues. As a Data Note, it allows the direct comparison of the performance of commercially available antibodies to amyloid-beta precursor protein to aid scientists in the Alzheimer’s Disease community to choose the most appropriate antibody for their studies.

 A committee of industry and academic representatives have endorsed the platform used, and the protocols used appear to be consistent with those in general use. The platform consisted of identification of a human cell line suitable for antibody characterization studies i.e., with adequate levels of the amyloid-beta precursor protein to generate a measurable signal. They found that the commercially available HAP1 cell line expressed the amyloid-beta precursor protein transcript at RNA levels above the average range of cancer cells, and therefore, it was a logical choice for their study. The matched isogenic knockout control cell line, *APP* KO HAP1 was used as an appropriate negative control. The final step was a series of antibody characterization procedures, limited to the most common research uses of antibodies. The interpretation of the results is left to the reader, with the study providing unbiased guidance. However, it gave some indication of the optimum dilutions of commercial antibodies. Two minor points to note. An ‘at a glance’ summary table encompassing the outcomes for each antibody would be useful. The IF figures are difficult to interpret using grayscale in this study, and it is not clear if any of the antibodies are suitable for IF. It might have been clearer to use the original colors.

 In conclusion, the study will be a useful resource when choosing the most appropriate antibody for study of the amyloid-beta precursor protein.

Are sufficient details of methods and materials provided to allow replication by others?

Yes

Is the rationale for creating the dataset(s) clearly described?

Yes

Are the datasets clearly presented in a useable and accessible format?

Yes

Are the protocols appropriate and is the work technically sound?

Yes

Reviewer Expertise:

Cell biology, DNA damage and repair, anti-cancer drug discovery.

I confirm that I have read this submission and believe that I have an appropriate level of expertise to confirm that it is of an acceptable scientific standard.

# Reviewer report: Reviewer response for version 2

**DOI:** 10.5256/f1000research.164529.r326944

**Published:** 2024-10-01

**Authors:** Jieya Shao

**Affiliations:** 1 Washington University in St Louis, St. Louis, Missouri, USA

In this article the authors systematically compared the performance and specificity of eleven commercially available anti-amyloid-beta precursor protein (APP) antibodies using three different experimental approaches (Western blot, immunoprecipitation, and immunofluorescence staining). This study is part of a large collaborative initiative to characterize commercial antibodies for different human proteins and offer unbiased guidance to researchers who can select the best antibodies for their specific experimental objectives based on their own interpretations. As a Data Note, this paper successfully achieved its goal with rigorously collected high-quality data, and the results are self-explanatory and highly useful for the research community.

 There are a few minor points to address. First, on page 5 under the methods-antibodies section, the authors mistakenly wrote anti-mouse secondary antibody twice one of which should be anti-rabbit. Second, on the same page under Western blot subsection, it may be helpful to briefly describe the method of protein normalization for equal loading. Third, on page 6 under Western blot subsection, the authors forgot to include the HAP1 APP KO cells in addition to WT.

Are sufficient details of methods and materials provided to allow replication by others?

Yes

Is the rationale for creating the dataset(s) clearly described?

Yes

Are the datasets clearly presented in a useable and accessible format?

Yes

Are the protocols appropriate and is the work technically sound?

Yes

Reviewer Expertise:

cancer biology, DNA damage response, DNA replication stress

I confirm that I have read this submission and believe that I have an appropriate level of expertise to confirm that it is of an acceptable scientific standard.

# Reviewer report: Reviewer response for version 2

**DOI:** 10.5256/f1000research.164529.r265022

**Published:** 2024-04-13

**Authors:** Amal Kaddoumi

**Affiliations:** 1 Auburn University, Auburn, Alabama, USA

The authors did not address any of my previous comments. They modified the title to become a "Guide to selecting.... etc." and the abstract to include that this work is part of a larger study to "characterize commercially available antibodies etc.". Yet, guidance, characterization, discussions, and recommendations are not provided. This paper is descriptive and provides figures produced from 3 different assays for 11 tested antibodies without giving any guidance for selection or recommendations.

Are sufficient details of methods and materials provided to allow replication by others?

No

Is the rationale for creating the dataset(s) clearly described?

Yes

Are the datasets clearly presented in a useable and accessible format?

Partly

Are the protocols appropriate and is the work technically sound?

Partly

Reviewer Expertise:

Neuropharmacology; Alzheimer's disease; CAA; blood-brain barrier

I confirm that I have read this submission and believe that I have an appropriate level of expertise to confirm that it is of an acceptable scientific standard, however I have significant reservations, as outlined above.

# Reviewer report: Reviewer response for version 1

**DOI:** 10.5256/f1000research.153179.r237210

**Published:** 2024-02-13

**Authors:** Jens-Ulrich Rahfeld

**Affiliations:** 1 Fraunhofer Institute for Cell Therapy and Immunology, Department Molecular Drug Biochemistry and Therapy, Weinbergweg 22, 06120, Halle, Germany

This manuscript "Identification of high-performing antibodies for amyloid-beta precursor protein for use in Western Blot, immunoprecipitation and immunofluorescence" fulfils the criteria for publication as a Data Note. The scientific topic - the immunological detection of the amyloid precursor protein APP in mammalian cells - is of current interest. On the one hand, this is based on research into the processes of neuronal development, homeostasis and interneuronal transmission and their connection to neurodegenerative diseases such as Alzheimer's disease. On the other hand, there are clear indications of increased expression of APP in malignant cells of various cancers.

 In this context, it is very important to have appropriate, carefully characterized tools for the detection of the APP protein.

 The manuscript contains a very impressive and well-designed overview and a head-to-head comparison of the performance of 11 commercially available antibodies from 5 different suppliers. It thus provides clear guidance for the selection of antibodies for a particular scientific or analytical question.

 Two defined and well-characterized cell lines were used for the analysis, whose characteristics with regard to APP expression were clearly confirmed in the course of the work.

 Minor Remark:

 In this manuscript, the authors do not evaluate the individual antibodies in terms of their different performance in the results section. It would be helpful for the reader to be provided with such a summary or an overview of the results obtained for each antibody. This could be provided in the form of an additional table, or as a supplement in Table 2 in additional columns.

Are sufficient details of methods and materials provided to allow replication by others?

Yes

Is the rationale for creating the dataset(s) clearly described?

Yes

Are the datasets clearly presented in a useable and accessible format?

Yes

Are the protocols appropriate and is the work technically sound?

Yes

Reviewer Expertise:

protein chemistry, antibody discovery, drug discovery, preclinical research, neurodegenerative diseases

I confirm that I have read this submission and believe that I have an appropriate level of expertise to confirm that it is of an acceptable scientific standard.

# Reviewer report: Reviewer response for version 1

**DOI:** 10.5256/f1000research.153179.r237217

**Published:** 2024-01-24

**Authors:** Amal Kaddoumi

**Affiliations:** 1 Auburn University, Auburn, Alabama, USA

In this report, the authors compared eleven APP commercial antibodies for Western blot, immunoprecipitation, and immunofluorescence using a standardized experimental protocol using HAP1-APP expressing cells (WT) and HAP1-APP KO cells. This report could be useful if the authors elaborated more on their recommendations based on their findings. I have the following comments:

- The authors should comment on the findings by clearly presenting their recommendations for which antibodies proved useful and for which assay. Also, they should comment on whether any of these antibodies could be used for the 3 assays, i.e., Western blot, immunoprecipitation, and immunofluorescence, or whether each assay requires one antibody but not the other.
- It’s unclear how many times each antibody was tested for validation/consistency. Is one time run/test enough to conclude?
- Each antibody specificity and species cross-reactivity should be reported.
- The antibodies showed 2 bands. Please comment on these 2 bands.
- Figure 3 presented binding in gray color; why is that? showing the images in their original staining colors (red, green, and blue for DAPI) would better clarify the binding. As presented, binding is not clear. Also, based on the results, which antibodies are recommended? As shown, the conclusions from Figure 3 are not clear.

Are sufficient details of methods and materials provided to allow replication by others?

No

Is the rationale for creating the dataset(s) clearly described?

Yes

Are the datasets clearly presented in a useable and accessible format?

Partly

Are the protocols appropriate and is the work technically sound?

Partly

Reviewer Expertise:

Neuropharmacology; Alzheimer's disease; CAA; blood-brain barrier

I confirm that I have read this submission and believe that I have an appropriate level of expertise to confirm that it is of an acceptable scientific standard, however I have significant reservations, as outlined above.

# Response

**Published:** 2024-04-01

**Authors:** Kathleen Southern

**Affiliations:** McGill University, Canada

Thank you Amal Kaddoumi for your comprehensive review of this study. A new version of this article has been submitted, to ensure transparency and prevent further misinterpretations. We trust that the refinements made meet your expectations, enhance comprehensibility and address any concerns you might have had. Please see our responses to your specific comments below. 

 To your first comment regarding providing antibody recommendations for each application, we’d like to clarify that the aim of the YCharOS initiative is not to recommend antibodies based on their performance under our standardized protocol. Furthermore, given that this article is formatted as a Data Note, it does not require the results to be discussed nor concluded. YCharOS is a public-private organization whose mission is to characterize antibodies for every human protein and deliver the research as a collective good for the scientific community. This study is to be used as a guide to help researchers, interested in studying amyloid-beta precursor protein or amyloid-beta proteins, select high-quality antibodies to enhance their experiments and prevent reproducibility and financial issues that occur when purchasing antibodies that don’t bind their target of interest properly. Moreover, as the antibodies are tested under one specific set of conditions, summarizing the performance of the antibodies or providing recommendations for each application would be valid only under the precise experimental setup and cell line used.

 That being said, we understand how the intention of our initiative may be misinterpreted. A new version has been submitted, to include modifications to the title, introduction and results&discussion section that ensure the authors goals are aligned and defined to all readers.

 To your second comment, the antibodies are tested in each application once. The amount of antibody provided by our industry partners (usually 100 µl) is enough to perform each application once. Furthermore, all antibodies tested per human protein are evaluated side-by-side under the same experimental set-up providing more transparency and accuracy of results, even when subjected to the experiment only once.

 For the same reasoning as in our reply to the first comment, the authors do not report antibody specificity or cross-reactivity. That being said, the following article written by Ayoubi et al., referenced in this article, describes the antibody performance criteria (Box 1) which may provide insights on the specificity of antibodies evaluated in this article (1)

 In response to your fourth comment, according to the antibody manufacturer catalogs, all antibodies tested in this study are designed to target the amyloid-beta precursor protein. According to literature, amyloid-beta precursor protein undergoes post-translational proteolytic cleavage that gives rise to amyloid beta peptides via proteases (2-4). The appearance of two bands in Western blot and immunoprecipitation may be attributed to post-translation activity, although this was not confirmed and is outside the scope of this study. Further experiments by experts in the amyloid beta protein family is required, using the high-quality antibodies that can be identified in this study.

 As described in the Figure legend of Figure 3, representative images are in gray due to the merged blue and red channel which generates a grayscale. The blue channel represents the nuclei stained with DAPI while the red channel represents the primary antibody staining. Therefore, the gray represents where the antibody is binding in the cell. Images of each channel can be found in the Dataset created for this study, in the *Underlying data* section.  

  

1. Ayoubi, R., et al. (2023). "Scaling of an antibody validation procedure enables quantification of antibody performance in major research applications." eLife **12**: RP91645
2. Müller UC, Zheng H: Physiological functions of APP family proteins. *Cold Spring Harb. Perspect. Med.*2012;2(2):a006288. 22355794 10.1101/cshperspect.a006288
3. Glenner GG, Wong CW: Alzheimer’s disease: initial report of the purification and characterization of a novel cerebrovascular amyloid protein. *Biochem. Biophys. Res. Commun.* 1984;120(3):885–890. 6375662 10.1016/S0006-291X(84)80190-
4. Kang J, Lemaire HG, Unterbeck A, et al.: The precursor of Alzheimer’s disease amyloid A4 protein resembles a cell-surface receptor. *Nature.* 1987;325(6106):733–736. 10.1038/325733a0
